# Macrophage Extracellular Traps Exacerbate Secondary Spinal Cord Injury by Modulating Macrophage/Microglia Polarization via LL37/P2X7R/NF-*κ*B Signaling Pathway

**DOI:** 10.1155/2022/9197940

**Published:** 2022-11-23

**Authors:** Chengyi Zhang, Dong Guo, Hao Qiao, Jie Li, Jiaxi Li, Yubing Yang, Su'e Chang, Fengtao Li, Dong Wang, Haopeng Li, Xijing He, Fang Wang

**Affiliations:** ^1^Department of Orthopaedics, The Second Affiliated Hospital, School of Medicine, Xi'an Jiaotong University, Xi'an, China; ^2^Department of Orthopaedics, Xi'an International Medical Center Hospital, Xi'an, China

## Abstract

Persistent inflammation in the secondary spinal cord injury (SCI) is an important reason for the failure of nerve repair, which is partly due to the continuous activation of local M1-like macrophage/microglia. It is reported that extracellular trap (ET) has been a new way of cell death, which can be released by macrophages and named macrophage extracellular trap (Met). Furthermore, it exists widely in the pathophysiological process of many diseases, but it has been rarely studied in the field of SCI. In this study, we constructed a spinal cord contusion model and assessed the function outcome of SCI rats. We used immunofluorescence, flow cytometry, and transmission electron microscope (TEM) to demonstrate the existence of Mets. Besides, some related experiments had also been employed to explore the relationship between Mets and M1 polarization of macrophage/microglia. We also performed Co-IP and Western blotting to reveal a new extracellular proinflammatory signal pathway. Finally, we made a linear regression analysis between the concentrations of specific markers of Mets in human serum and ASIA scores. Briefly, our results suggested that macrophages infiltrated in SCI area could induce macrophage/microglia to differentiate into M1-like cells by releasing Mets, which may be achieved partly through LL37-P2X37-NF-*κ*B signal pathway. However, limiting Mets could effectively inhibit M1 polarization and promote function recovery. In addition, the concentrations of Met related proteins in human serum showed high correlation with ASIA scores and could be applied to reflect the severity of SCI. In conclusion, Mets may be a new target for SCI therapy and a promising index for SCI assessment.

## 1. Introduction

Persistent inflammation in the stage of secondary spinal cord injury (SCI) is one of the important reasons for the failure of nerve regeneration [1, 2]. The polarization of macrophage/microglia makes them play a double-edged role in the process of nerve regeneration and complicates the repair of SCI [3]. According to the current theory, classically-activated M1-like macrophage/microglia cells are generally considered to promote SCI. On the other hand, alternatively-activated macrophage/microglia cells have neuroprotective properties and play an anti-inflammatory role through a variety of mechanisms, such as the production of anti-inflammatory cytokines and enhanced phagocytosis to clear cell fragments [2–4]. Macrophages are one of the important components of the innate immune system. They have the functions of phagocytosis, antigen presentation, and secretion of various cytokines and play a critical role in physiological processes such as inflammation, defense, repair, and metabolism. Meanwhile, they are also a key factor to maintain our body stability [4, 5]. In addition, Macrophages have the characteristics of high plasticity, local-tissue function specificity, and abnormal differentiation induced by inflammatory factors [4]. Our previous study found that one week after SCI, most of the immune cells infiltrated in the injury center were bone marrow-derived macrophages (BMDMs) and most of them existed in the form of M1-like macrophages, which may be one of the reasons for the progressive aggravation of SCI [6, 7]. Nowadays, most of the studies on the regulation of macrophage/microglia polarization focus on intracellular signaling pathways, such as JAK/STAT [8]. However, there are few reports on the molecular pathway of the extracellular regulation of macrophage/microglia polarization.

The generation of extracellular trap (ET) has been considered a new path to cell death [9]. ET were originally discovered in the study of the bactericidal mechanism of neutrophils and was described as an extracellular reticular structure (Net), which was composed of DNA and proteins and released by neutrophil [10]. And it can be degraded by DNase I [11–13]. Besides, some studies found that macrophages can also produce similar structures named macrophage extracellular trap (Met) [14]. Although Met has been studied in a few fields, such as infection [15–17] and acute renal injury [18, 19], it is not clear whether it is involved in SCI and its role in SCI.

In this study, we demonstrated the existence of Mets in SCI. We also studied the relationship between Met and macrophage/microglia polarization in the injured area. Besides, we explored its potential mechanism in the progression of persistent inflammation after SCI and found a new extracellular signal pathway to promote the inflammatory response. In addition, we briefly analyzed the relationship between the specific markers of Mets in human serum and the stage of SCI and ASIA score. All in all, our study aims to provide a new target for the treatment of local inflammation of secondary SCI and a new index for clinical evaluation of the severity of SCI.

## 2. Materials and Methods

### 2.1. SCI Model and Grouping

96 experimental rats were provided by the Experimental Animal Center of Xi'an Jiaotong University. All of them were healthy-adult male Sprague-Dawley (SD) rats (170-220 g). This study was approved by the Ethics Committee of the Second Affiliated Hospital of Xi'an Jiaotong University. The treatment of these animals conformed to the standards in the Guidelines for Laboratory Animal Care and Use of the National Institutes of Health (NIH).

In this study, we used the modified Allen method to make the spinal cord injury (SCI) model [20]. The experimental design and grouping are shown in Figure S2. Procedure: The rats were anesthetized by intraperitoneal injection of 1% pentobarbital solution (40 mg/kg). After skin preparation and T9-T11 vertebras exposure, laminectomy was performed at the T10 vertebral level to expose the thoracic spinal cord. After that, the rats were placed on the table of the NYU/MASCIS Impactor (Model II; Rutgers, USA) with the back facing upward, and a 10 g impact rod was placed vertically above the exposed spinal cord. The lower end of the impact rod was adjusted to be 25 mm from the surface of the spinal cord, and then it was released to strike it against the exposed spinal-cord surface to make the spinal-cord contusion model. After the strike, the hematoma was rapidly generated on the surface of the spinal cord. Besides, tail-flick reflex and transient twitch of the rat's hind limbs occurred 2-5 seconds later, indicating that the modeling was successful. In addition to the Sham group, the rats in the other two groups underwent artificial urination (2 times per day) after the operation, and penicillin G (20 U/kg) was injected subcutaneously to prevent postoperative infection until the hematuria disappeared.

In grouping, all the 96 experiment rats were randomly divided into three groups: Sham group (*n* = 32), SCI group (*n* = 32), and DNase I group (*n* = 32). In Sham group, laminectomy was performed only. In SCI group, spinal cord contusion was induced by a 10-g rod falling from the height of 25 mm after laminectomy [21]. In DNase I group, rats in this group were injected with DNase I (dilate in saline; Roche; 5 mg/kg) via tail vein immediately after SCI was induced. All these rats were treated with warmth, rehydration, analgesia, and anti-infection after the operation. SCI: Spinal Cord Injury; DNase I: Deoxyribonuclease I.

### 2.2. Tissue Extraction and Sectioning

In tissue extraction, at 7 days after the operation, 26 rats in each group were euthanized deeply. After the PBS was perfused into the heart, 10-millimeter-long spinal cord tissue was taken from the center of the injury.

In frozen slice production, the tissue blocks were fixed in 4% paraformaldehyde and placed in sucrose solution for gradient dehydration. Then after being frozen and embedded, they were placed on a constant cold frozen slicer for continuous crosscutting. Slice thickness is 10 *μ*m. Then, the slices were put in a slicing box and stored in a refrigerator at -80°C.

### 2.3. Behavioral Assessment

Basso-Beattie-Bresnahan (BBB) scale, inclined plate test, and footprint analysis were used to assess the motor function of rats in the three groups. In BBB scale, the muscle strength and joint activity of the rats' limbs in the three groups were observed and scored 0, 1, 7, 14, 21, and 28 days after the operation, respectively. The score ranges from 0 to 21. In inclined plate test, the maximum angles of the inclined plate with rats staying on it for 10 seconds was recorded at 0, 1, 7, 14, 21, and 28 days after the operation, respectively [21]. Footstep analysis, immerse the hind feet of rats in ink, lay one-meter-long absorbent paper, and then make them walk along the absorbent paper freely 7 days after the operation. After that, measure the step lengths, the distance between feet, and the rotation angle of the hind feet of rats from all three groups. The three evaluation methods above were independently evaluated by two evaluators who know nothing about this study. The final result was the average of the two evaluation results.

### 2.4. Electrophysiological Assessment

Electromyographic (EMG) monitor was used to observe the recovery of nerve conduction of rats in the three groups. The stimulation electrode was placed in the motor area of the rat's cerebral cortex and the recording electrode was placed on the contralateral calf gastrocnemius. The rats in each group were given the same electrical stimulation and the motor evoked potentials (MEPs) were recorded 7 days after the operation.

### 2.5. H&E Staining and Nissl Staining

H&E staining was used to observe the general tissue morphology and cell morphology in the injured area. Steps were as follows: the frozen slices were stained with hematoxylin staining solution for 5 min, differentiated in differentiation solution for 2 s, and dealt with the return-to-blue solution for 10 s. Rinse with distilled water for 20 s between each operation to remove the liquid from the previous operation. When all these steps were done, the slices were dehydrated (80%, 90%, 95%, and 100% alcohol; 3 s), made transparent (Xylene I, II, 1 min), and finally sealed with neutral gum. Then, they were observed and photographed under the optical microscope and the ratio of cavity area to lesion center area in each group was calculated.

Nissl staining was used to observe the remnant neurons. Steps were as follows: the slices were placed in a dyeing vat with Cresyl violet Stain solution, immersed in a 56°C incubator for 1 h, and then washed with deionized water. After differentiation (Nissl Differentiation solution, 1 min), gradient dehydration, transparency, and sealing, the tissue slices were observed and photographed under the optical microscope, and then the number of positive cells in each group was calculated.

### 2.6. TUNEL Staining

TUNEL staining was used to stain and count apoptotic cells. According to the manufacturer's instructions, the slices were stained by using an in-situ cell death detection kit (DeadEnd™ Fluorometric TUNEL System, Promega, USA). Briefly, DNase-free proteinase K was diluted 1,000-fold to 20 *μ*g/ml of working solution with 10 mM Tris HCl solution for the permeabilization of the slices for 20 min. After washing with PBS, slices were stained with the prepared TUNEL detection solution and incubated at 37°C for 60 min in dark. With repeated washing using PBS, the next step was staining the nucleus with DAPI. After that, the positive cells were observed under a fluorescence microscope (IX71; Olympus, Tokyo, Japan). Five visual fields were randomly selected from each slice, and the average value was recorded to quantitatively identify the number of apoptotic cells to count the number of positive cells.

### 2.7. Immunofluorescence Staining

CitH3, CD68, Iba-1, CD16/32, and CD206 markers in spinal cord tissues were stained to count and analyze the subcellular localization of Mets, M1, and M2-like cells. Based on the previous research, we know that CitH3 and CD68 are used to label Mets, Iba-1 is used to label macrophage/microglia, CD16/32 is used to label M1-like cells, and CD206 is used to label M2-like cells. DAPI is used to label extracellular DNA and nucleus. 6 frozen slices from each group were used for CitH3 and CD68 staining. In this experiment, we used a TSA detection kit (TSAPLus fluorescent double-staining kit; Servicebio) for fluorescence double staining. The experimental steps were carried out strictly following the manufacturer's instructions. Briefly, 6 frozen slices from each group were fixed, infiltrated, blocked, and then incubated with the corresponding primary antibody at 4°C overnight, followed by the addition of the secondary antibody of FITC-conjugated goat anti-rabbit IgG (1 : 200; Servicebio) for 50 min of incubation at room temperature. After washing with PBS, TSA was added dropwise for a subsequent 10 min of incubation at room temperature in dark. The second primary antibody of rabbit anti-histone H3 (citrulline R2+ R8 + R17) polyclonal antibody (1 *μ*g/mL; Abcam) was added dropwise after microwave treatment, followed by the addition of the secondary antibody of Cy3-conjugated goat anti-rabbit IgG (1 : 200; Servicebio) after incubation overnight. After that, the nuclei was restained with DAPI, the fluorescence intensity was analyzed and counted with ImageJ software (version 1.8.0; National Institutes of Health, Bethesda, MD, USA). Another 6 frozen slices were taken from each group for the staining of Iba-1, CD16/32 and CD206 according to the same steps mentioned above. CitH3: Citrullinated Histone H3. The primary antibodies used in the experiment were as follows: Rabbit anti-histone H3 (citrulline R2 + R8 + R17) polyclonal antibody (1 *μ*g/mL; Abcam), Rabbit anti-CD68 polyclonal antibody (0.5 *μ*g/ml; Abcam), Rabbit anti-Iba1 monoclonal antibody (1 : 500; Abcam), Mouse anti-CD16/32 (1 : 200; Abcam), rabbit anti-CD206 polyclonal antibody (1 : 1000; Proteintech), and corresponding secondary antibodies were FITC-conjugated goat anti-rabbit IgG (1 : 200; Servicebio), Cy3-conjugated goat anti-rabbit IgG (1 : 200; Servicebio), Alexa Fluor® 488-conjugated goat anti-rabbit IgG (1 : 200; Servicebio), and Cy3-conjugated goat anti-mouse IgG (1 : 200; Servicebio).

### 2.8. Flow Cytometry

Flow cytometry was used to analyze the proportion of Mets forming cells in the injured area. Steps were as follows: the spinal cord tissue was cut into pieces in 4°C PBS, centrifuged, digested, and recentrifuged. After that, 200 *μ*L PBS was added and the tissue homogenate was resuspended in the filter. Then, 2 *μ*L Rabbit anti-histone H3 (citrulline R2 + R8 + R17) polyclonal antibody (1 *μ*g/mL; Abcam) and Mouse anti-CD68 monoclonal antibody (1 : 100; Abcam) were added to the resuspended cells. The mixture was incubated 30 min on ice in dark and then it was centrifuged and the supernatant was removed. Then, the precipitation was washed with 500 *μ*L PBS for 3 times, and 200 *μ*L PBS was added to resuspend. Afterward, add 2 *μ*L secondary antibody of Donkey Anti-Rabbit IgG H&L (Alexa Fluor 647) (1 : 2000; Abcam), and Goat Anti-Mouse IgG H&L (Alexa Fluor 405) (1 : 2000; Abcam), respectively, and incubate on ice for 30 min without light. Repeat centrifuging and washing for 3 times. The resorted cells were put into a flow cytometer (MACSQuant^®^ to Analyzer 10; Miltenyi Biotec, German) for flow detection. In the scatter plot of flow cytometry, CD68+ and CitH3+ double-positive cells were considered to be Mets forming cells.

### 2.9. Western Blotting

Western blotting was used to detect the expression of corresponding proteins. GAPDH was used as a loading control. Steps were as follows: in protein extraction, spinal cord tissues from the three groups were removed from the refrigerator at -80°C and placed in centrifugal tubes on ice. Then, RIPA lysate containing PMSF was added inside (1 g spinal cord tissue was lysed with 10 mL RIPA; RIPA: PMSF = 100 : 1). The mixture was fully homogenized by a low-temperature homogenizer and centrifuged (4°C; 12000 rpm; 15 min). Take out the supernatant. Use BCA to detect the protein concentration. After that, 20 *μ*g protein and the same amount of Marker were injected into SDS-PAGE gel pores for electrophoresis and then electroporated through the PVDF membrane. Later, it was sealed with 3% fresh skimmed milk powder at room temperature for 2 h and then the primary antibody was added and incubated at 4°C overnight. The membrane was washed with TBST (*n* = 3; 5 min per time), and then the second antibody of Goat Anti-Rabbit IgG H&L (HRP) (1 : 2000; Abcam) was added and incubated for 1 h. The membrane was washed again. Finally, it was detected by ECL chemiluminescence and the relative expression of the target proteins was calculated. The primary antibody used in the experiment: rabbit anti-LL37 polyclonal antibody (1 : 1000; eBioscience) rabbit anti-CD68 polyclonal antibody (0.5 *μ*g/ml; Abcam), Rabbit anti-Iba1 monoclonal antibody (1 : 1000; Abcam), Rabbit anti-CD16 monoclonal antibody (1 : 1000; Abmart, Shanghai), Rabbit anti-iNOS monoclonal antibody (1 : 1000; Abcam), rabbit anti-CD206 polyclonal antibody (1 : 1000; Proteintech), rabbit anti-arginase (Arg)-1 polyclonal antibody (1 : 1000; ProteinTech), rabbit anti-P2X7R polyclonal antibody (1 : 1000; Abcam), rabbit anti-NFkB-p65 polyclonal antibody (1 : 1000; eBioscience), and Rabbit anti-Phospho-NFkB-p65 monoclonal antibody (1 : 1000; eBioscience).

### 2.10. TEM

A transmission electron microscope (TEM) was used to explore the ultrastructure and spatial distribution of Mets. The tissue was prefixed with 3% glutaraldehyde and 1% osmium tetroxide. Then it was dehydrated stepwise with acetone solution. After that, it was permeated with anhydrous acetone+epoxy resin, embedded with an embedding agent, and polymerized by heating. The embedded block was sliced on the ultrathin slicing machine. Each slice was 50 nm thick. The slices were stained with uranium acetate for 10 min and then stained with lead citrate dye for 2 min at room temperature. The stained slices were observed under TEM (H-600; Hitachi, Japan).

### 2.11. RT-qPCR

RT-qPCR was used to detect the gene expression of corresponding markers of M1 and M2-like cells. Procedures were as follows: take out the spinal cord tissue, add 1 mL Trizol, and fully homogenize, mix with 0.2 mL chloroform, centrifuge (4°C; 12000 rpm; 15 min) after static placement of 5 min, and obtain the supernatant. Then, add precooled isopropanol (equal volume), mix well, centrifuge (4°C; 12000 rpm; 10 min), and discard the supernatant. Add precooled ethanol (75%; 1 mL), and then centrifuge again (4°C; 12000 rpm; 5 min). Discard the supernatant and dissolve the precipitate in 20 *μ*L DEPC. Reverse transcription of the total RNA to cDNA. The conditions were as follows: 25°C, 10 min; 48°C, 30 min; and 95°C, 5 min. Then place on ice and add PCR ingredients in turn following the manufacturer's instructions. Conditions setting: predenaturation for 15 min; denaturation (95°C; 15 s); and annealing at 56°C, extension (68°C, 30 s). Finish a total of 40 cycles of amplification. After amplification, read out the CT value and quantitatively analyze it by the CT method [22]. The sequences of primers are shown in Table 1.

### 2.12. Co-IP

Co-immunoprecipitation (Co-IP) was used to explore the interaction between LL37 and P2X7R. LL37 is an antimicrobial peptide and participates in the formation of Mets. P2X7R, an ATP-gated ion channel, is highly expressed on the cell membrane of microglia/monocyte. P2X7R: P2X purinoreceptor 7. Procedures were as follows: take out the tissue and split it on ice (the same as Western blotting). Fully homogenize and centrifuge (14000 rpm; 4°C; 10 min), and then transfer the supernatant into the centrifuge tube. After that, add 500 *μ*L lysate, add 5 *μ*g rabbit anti-LL37 polyclonal antibody (1 : 1000; eBioscience), use nonspecific homologous antibody as control, and mix all the additives gently at 4°C overnight. Centrifuge (12000 rpm; 1 min) and then discard the supernatant. Add 0.5 mL RIPA lysate and shake it gently. Then centrifuge again and remove the supernatant. Repeat it 3 times. Add 40 *μ*L 1X SDS-PAGE electrophoresis sample buffer, treating at 100°C for 5 min. Then start the Western blotting (details were shown in Western blotting).

### 2.13. Detection of Human Serum Markers and ASIA Scale

The experimental design and grouping are shown in Figure S2. Table S2 shows the inclusion and exclusion criteria of subjects. In this study, 8 healthy volunteers and 24 patients at different SCI stages were screened and recruited. Then, they were divided into 2 groups: volunteer group (*n* = 8), SCI group (*n* = 24) (including patients in acute stage (*n* = 8), subacute stage (*n* = 8), and chronic stage (*n* = 8)).

Detection of human serum markers, 5 mL venous blood from each person was collected and placed in the anticoagulant tube. Centrifugate (1500 rpm) at 4°C for 5 min and collect the serum. The concentrations of cf-DNA, CD68, and CitH3 in the serum of the four groups were detected by using three kinds of biological detection kits: cf-DNA detection kit (Quant-iT™ PicoGreen™ dsDNA Assay Kits and dsDNA Reagents; eBioscience), CD68 detection kit (Human CD68 ELISA Kit; Abcam), and CitH3 detection kit (Citrullinated Histone H3 (Clone 11D3) ELISA Kit; Cayman Chem). The experimental steps were carried out strictly following the manufacturer's instructions.

Evaluate and record the ASIA (American Spinal Injury Association) scores of each member in these groups. The degree of neurological injury was graded by the International Standard of Neurological Classification of Spinal Cord Injury (American Spinal Injury Association, ASIA, 2011 version) [23], and the severity of spinal cord injury was quantified by ASIA motor and sensory scores.

ASIA damage classification [23] was used to group the injury degree of the patients. Grade A is complete injury, that is, no sensory or motor function is retained in the Sellar region dominated by S4 ~ S5. Grade B is incomplete sensory injury, that is, below the injured nerve plane, including the Sellar region dominated by S4 ~ S5, there is no motor function but sensory function was preserved, and there is no motor function reservation of more than 3 segments below the nerve injury plane of any side of the body. Grade C is incomplete motor injury, that is, motor function is preserved below the injured nerve plane, and the muscle strength of more than half of the key muscles below the injured nerve plane is less than grade 3. Grade D is incomplete motor injury, but the degree of the injury is lighter than Grade C, that is, the motor function is preserved below the injured nerve plane, and the muscle strength of at least half of the key muscles below the injured nerve plane is more than or equal to grade 3. Grade E is normal, that is, the motor function and sensory function of all segments are normal. When the patient had neurological dysfunction in the past, the grade is still regarded as Grade E.

ASIA sensory score [23] was used to quantify the severity of sensory impairment. The sensory score is the total score of acupuncture and the light tactile sensation of each skin segment on both sides of the body. Normally, the score of each state is 2 points, and there are 28 key points on each side, so the total scores of the acupuncture and the light tactile sensation on one side are 56 points, respectively. Therefore, the full score of body sensation on one side is 112 points, so the full score of normal sensory function is 224 points.

ASIA motor score [23] was used to quantify the severity of motor function injury. The motor scoring method is to examine the muscle strength of 10 groups of the key sarcomeres on the left and right sides of the body. According to the rating of muscle strength, Grade 0-5 corresponds to 0-5 points, so the full score of normal motor function is 100. The higher the score, the better the muscle function. On the contrary, the lower the score, the worse the muscle function.

Finally, we analyzed the relationship between the concentration levels of cf-DNA, CD68, and CitH3 and ASIA scores. ASIA scores were independently evaluated by three evaluators who know nothing about this study. The final result was taken as the average value of the three results.

In addition, the collection of human blood samples has obtained the consent of all volunteers, and the operation of human blood samples has been approved by the Ethics Committee of the Second Affiliated Hospital of Xi'an Jiaotong University.

### 2.14. Statistical Analysis

All the data in this study were statistically analyzed by SPSS v18.0.0 (IBM, NY, USA). Double-tailed *t*-test analysis was used for the comparison of two groups of quantitative data. Single-factor analysis of variance (ANOVA) was used for multigroup comparison and the Bonferroni method was used for pairwise comparison [24]. Chi-square test analysis was used for the comparison of two groups of qualitative data. The experimental charts were drawn by GraphPad Prism 8 (GraphPad, La Jolla, CA, USA). All experimental data were expressed as mean ± standard deviation (Mean ± SD). *P* < 0.05 was considered statistically different.

## 3. Results

**Figure 1:**
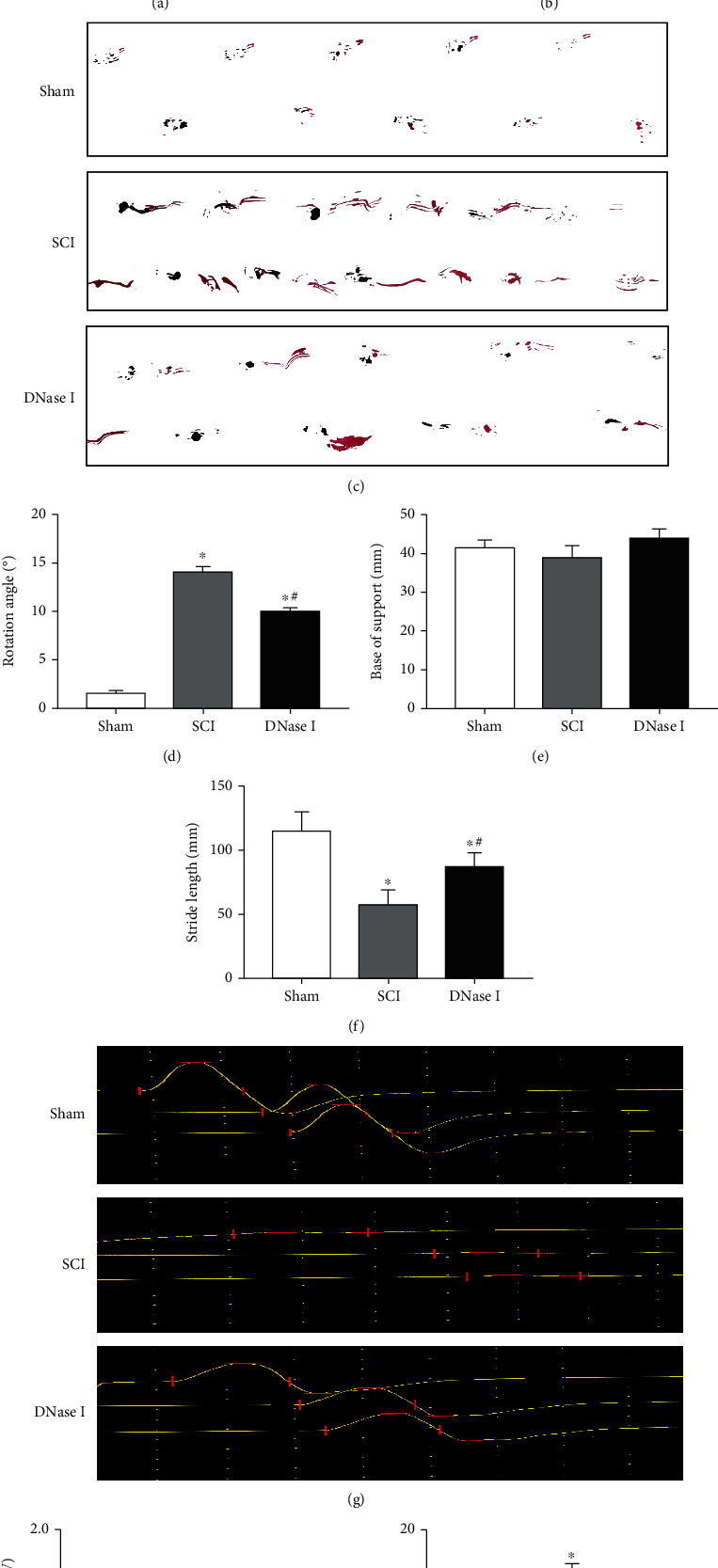
Limiting the number of Mets promotes the recovery of motor function and nerve conduction. (a) BBB scores of the Sham, SCI, and DNase I groups at 0, 7, 14, 21, and 28 days after the operation, respectively. Data are exhibited as Mean ± SD (*n* = 6).  ^∗^*P* < 0.05 vs. Sham group; #*P* < 0.05 vs. SCI group (one-way analysis of variance followed by the least significant difference test). SCI: spinal cord injury; BBB scale: Basso-Beattie-Breshman Locomotor Rating Scale. (b) Inclined plane test results of the three groups at 0, 7, 14, 21, and 28 days after the operation, respectively. Data are displayed as Mean ± SD (n = 6).  ^∗^P < 0.05 vs. Sham group; #P < 0.05 vs. SCI group. (c) Footprint map of three groups. (d–f) Representative bar graphs of rotation angle, the base of support, and stride length, respectively. Compared with those in the SCI group, the rotation angle and stride length in DNase I group were significantly improved. Data are shown as Mean ± SD (n = 6).  ^∗^P < 0.05 vs. Sham group; #*P* < 0.05 vs. SCI group. (g) The waveform of MEP in Sham, SCI, and DNase I groups. Horizontal lines indicate the positions of peaks and troughs. The vertical lines split the periods of waves. (h–i) Typical bar graphs of MEP amplitude and latency in three groups, respectively. (h) The MEP amplitude in DNase I group is larger than the SCI group and similar to the Sham group. (i) The MEP latency in DNase I group is shorter than the SCI group and similar to the Sham group. Data are presented as Mean ± SD (n = 6).  ^∗^P < 0.05 vs. Sham group; #*P* < 0.05 vs. SCI group. MEP: motor evoked potentials.

Limiting the number of Mets can promote the recovery of motor function and nerve conduction after spinal cord injury (SCI). Primarily, we limited the number of Mets by tail-vein injection of DNase I after SCI. To intuitively understand the effect of the limitation of Mets on the recovery of motor function and nerve conduction, our study evaluated it from two aspects. BBB scale, inclined plate test, and footprint analysis were used to evaluate the recovery of motor function (Figures 1(a)–1(f)). Electromyography was used to evaluate the recovery of nerve conduction (Figures 1(g)–1(i)). On the BBB scale, compared with those in the SCI group, rats in the DNase I group got higher BBB scores during the 28-day observation. And the improvement of the BBB scores in the DNase I group was also better than that in the SCI group during the observation. There was a significant difference in the change of BBB scores between DNase I and SCI groups (*P* < 0.05) (Figure 1(a)). The results of the inclined plate test in the three groups showed the same trend (Figure 1(b)). In addition, we found that the stride length, rotation angle, and base of support in the DNase I group were significantly improved, compared with those in the SCI group (Figures 1(c)–1(f)). All these results proved that the limitation of Mets can promote the recovery of motor function after SCI. Moreover, electromyography showed that the MEP latency and amplitude in the DNase I group were better than those in the SCI group but lower than those in the Sham group. The differences among the three groups were statistically significant (*P* < 0.05) (Figures 1(g)–1(i)). This suggested that limiting the number of Mets can promote the recovery of nerve conduction after SCI.

**Figure 2:**
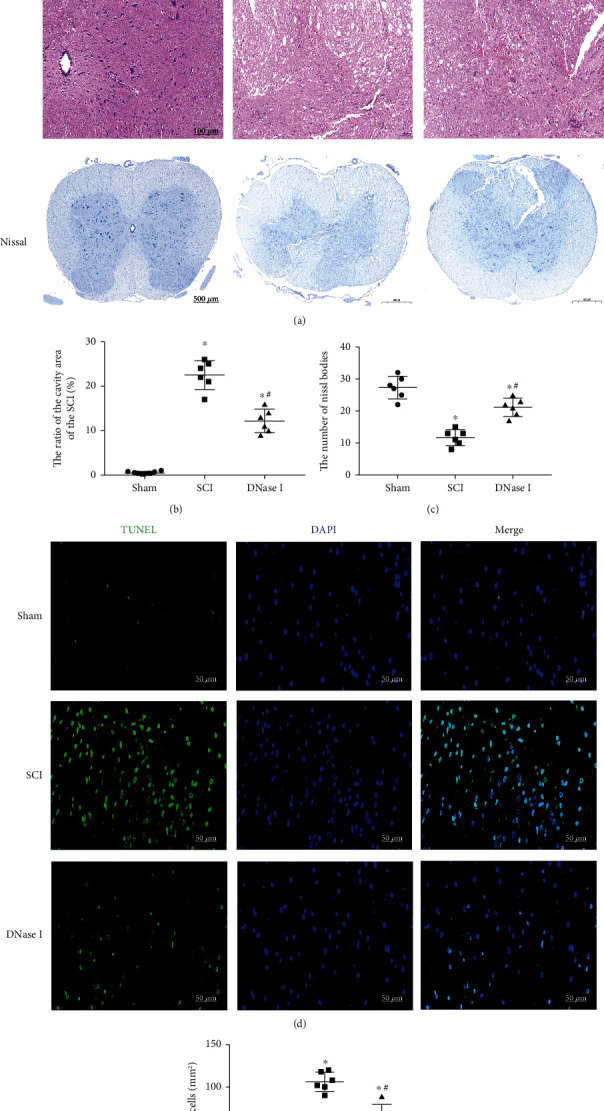
Limiting the number of Mets improves the pathological environment of the injured area. (a) H&E and Nissl staining of the injured area in Sham, SCI, and DNase I groups at 7 days after the operation. The black boxes point to the local tissue magnification. Scale bars of H&E staining: 500 *μ*m, 100 *μ*m. Scale bar of Nissl staining: 500 *μ*m. H&E staining: hematoxylin-eosin staining. (b) Representative scatterplot of the ratio of the cavity area. The ratio of the cavity area in the DNase I group is lower than that in the SCI group but higher than that in the Sham group. Data are exhibited as Mean ± SD (n = 6).  ^∗^P < 0.05, vs. Sham group; #P < 0.05 vs. SCI group. (c) Scatterplot of the number of Nissl bodies. The number of Nissl bodies in the DNase I group is higher than that in the SCI group but lower than that in the Sham group. Data are shown as Mean ±SD (n = 6).  ^∗^*P* < 0.05 vs. Sham group; #*P* < 0.05 vs. SCI group. (d) TUNEL staining of the injured area in the Sham, SCI, and DNase I groups at 7 days after the operation. Dead cells (green), nuclear (blue). Scale bar: 50 *μ*m. (e) Typical scatterplot of the fluorescence area of TUNEL^+^ cells. The fluorescence area of TUNEL^+^ cells in the DNase I group is lower than that in the SCI group but higher than that in the Sham group. Data are presented as Mean ± SD (n = 6).  ^∗^*P* < 0.05 vs. Sham group; #*P* < 0.05 vs. SCI group.

Limiting the number of Mets can improve the local pathological environment of the injury. Although both the behavior and electrophysiological evaluation reflect the positive effect of the limitation of Mets on the repair of SCI, we still need to evaluate the improvement of the local pathological environment. In H&E staining, compared with the SCI group, the tissue in the DNase I group showed less infiltration of inflammatory cells and almost no syringomyelia formation (Figures 2(a), 2(b)). Thus, it can be seen that using DNase I to limit the number of Mets can significantly alleviate the local inflammation of the injury. Meanwhile, Similar comparison results can be seen in Nissl staining and TUNEL staining. In Nissl staining, there were more Nissl bodies in the DNase I group than those in the SCI group, which suggests that limiting the number of Mets can significantly improve the damage of neurons in the injured area (Figures 2(a), 2(c)). In TUNEL staining, the limitation of Mets significantly reduced the number of apoptosis cells in the injured area (Figures 2(d), 2(e)). In conclusion, limiting the number of Mets can improve the local pathological environment of the injury.

### 3.1. Macrophages in the Injury Can Produce Mets

**Figure 3:**
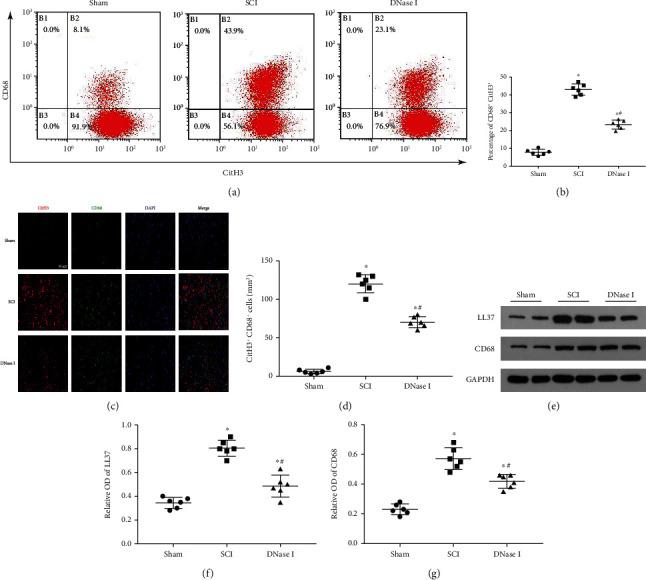
Macrophages in the injured area produce Mets. (a) Representative flow plots show the relative cell composition labeled by CD68 and CitH3 in the injured area of each group 7 days after the operation. CitH3: Citrullinated Histone H3. (b) Scatterplot of the percentage of CitH3^+^ CD68^+^. The percentage of CitH3^+^ CD68^+^ in the DNase I group is lower than that in the SCI group but higher than that in the Sham group. Data are presented as Mean ± SD (*n* = 6).  ^∗^*P* < 0.05 vs. Sham group; #*P* < 0.05 vs. SCI group. (c) Typical fluorescence staining images of CitH3 (red) and CD68 (green) double-positive cells in the injured area of each group at 7 days after the operation. Nuclear was labeled with DAPI (blue). Scale bar: 50 *μ*m. (d) Scatterplot of the area of CitH3^+^ CD68^+^ cells in three groups. The percentage of the area of CitH3^+^ CD68^+^ cells in the DNase I group is lower than that in the SCI group but higher than that in the Sham group. Data are presented as Mean ± SD (*n* = 6).  ^∗^*P* < 0.05 vs. Sham group; #*P* < 0.05 vs. SCI group. (e) Typical immunoblots of LL37 and CD68 in the injured area of each group at 7 days after the operation. GAPDH was used as a loading control. (f, g) Scatterplot of the relative OD of LL37 and CD68 in each group. The relative OD of LL37 and CD68 in the DNase I group is lower than that in the SCI group but higher than that in the Sham group. A comparison of relative OD of CD68 among three groups shows analogous results. Data are presented as Mean ± SD (*n* = 6).  ^∗^*P* < 0.05 vs. Sham group; #*P* < 0.05 vs. SCI group. OD: Optical Density.

To demonstrate the existence of Mets in the injured area, we performed immunofluorescence staining on spinal cord tissue slices 7 days after SCI. From Figures 3(c), 3(d), we could see that the fluorescence intensity of CitH3 and CD68 increased significantly in the SCI group, and there was a colocalization phenomenon. However, compared with the SCI group, the fluorescence intensity decreased significantly in the DNase I group (*P* < 0.05). It is suggested that the macrophages in the injury can produce Mets under natural pathological conditions, and these Mets can be degraded by DNase I. Flow cytometry detection of the tissue from the three groups showed that DNase I could significantly reduce the proportion of CitH3+ and CD68+ double-positive cells in the injured area, which confirmed the existence of Mets in the injury from another side (Figures 3(a), 3(b)). In addition, we used Western blotting to analyze CD68 and LL37 qualitatively and quantitatively. The results also demonstrated the existence of Mets (Figures 3(e)–3(g)). Under TEM, we could see the extracellular DNA structure originated from macrophages and extended outside the cell membrane, which demonstrated that the ETs come from macrophages (Figure S1).

### 3.2. Limiting the Number of Mets Can Promote M2 Polarization of Macrophage/Microglia in the Injury

**Figure 4:**
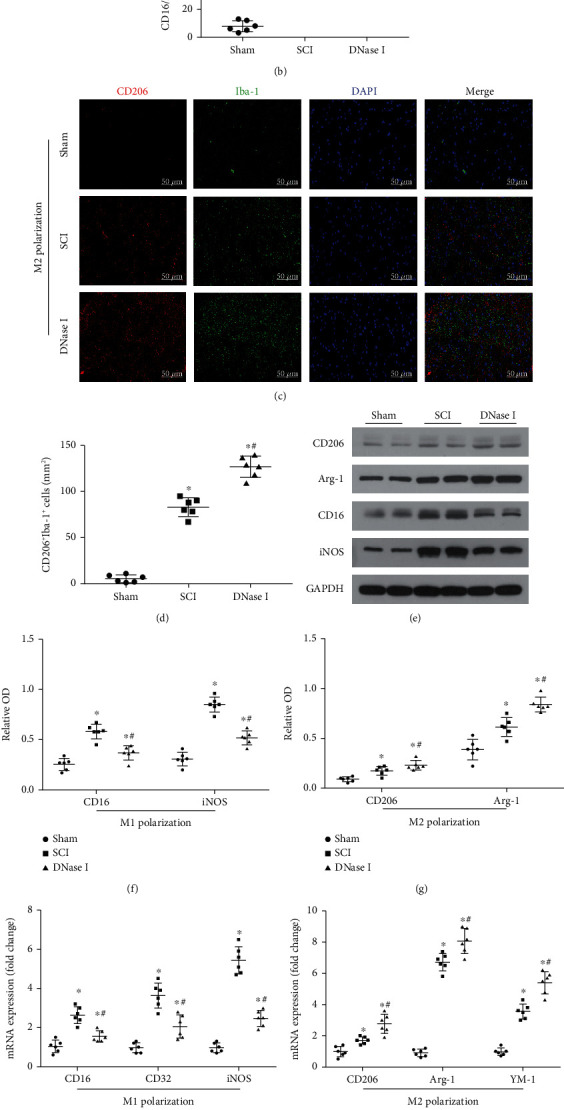
Limiting the number of Mets promotes M2 polarization of macrophage/microglia in the injured area. (a) Representative fluorescence staining images of CD16/32 (red) and Iba-1 (green) double-positive cells in the injured area of each group at 7 days after the operation. Nuclear (blue). Scale bar: 50 *μ*m. (b) Scatterplot of the area of CD16/32^+^ Iba-1^+^ cells in each group. The area in DNase I group is lower than that in the SCI group but higher than that in the Sham group. (c) Typical images of CD206 (red) and Iba-1 (green) double-positive cells in the injured area of each group at 7 days after the operation. Scale bar: 50 *μ*m. (d) Scatterplot of the area of CD206^+^ Iba-1^+^ cells in each group. The comparison results of the area of the three groups are the same as b. (e) Representative immunoblots of CD206, Arg-1, CD16, and iNOS in the injured area of each group at 7 days after the operation. (f) Scatterplot of the relative OD of CD16 and iNOS in each group. The relative OD of CD16 and iNOS in the DNase I group is lower than that in the SCI group but higher than that in the Sham group. (g) Scatterplot of the relative OD of CD206 and Arg-1 in each group. The relative OD of CD206 and Arg-1 in the DNase I group is considerably higher than that in the SCI group. (h) Scatterplot of CD16, CD32, and iNOS mRNA expression in each group using RT-qPCR. RT-qPCR: Real Time-quantitative PCR. (i) Scatterplot of CD206, Arg-1, and YM-1 mRNA expression in each group. (j) Scatterplot of the cytokine concentrations of IL-1*β* and IL-10 in each group. Data from all the above scatterplots are presented as *Mean* ± *SD* (*n* = 6).  ^∗^*P* < 0.05 vs. Sham group; #*P* < 0.05 vs. SCI group (one-way analysis of variance followed by the least significant difference test).

To analyze the relationship between Mets and macrophage/microglia polarization in the injured area, we used immunofluorescence staining to stain macrophage/microglia marker (Iba-1), M1-like cell marker (CD16/32), and M2-like cell marker (CD206) in spinal cord tissue slices at 7 days after SCI. In M1 polarization, the fluorescence intensity of Iba-1 and CD16/32 in the DNase I group was significantly higher than that in the Sham group but lower than that in the SCI group (*P* < 0.05). It is suggested that a large number of M1-like cells infiltrate locally at 7 days after SCI and using DNase l to degrade the Mets can reduce the number of M1-like cells infiltrated in the injured area (Figures 4(a), 4(b)). In M2 polarization, we found that the fluorescence intensity of Iba-1 and CD206 in the DNase l group was significantly higher than that in the SCI group (*P* < 0.05), indicating that limiting the number of Mets can increase the number of M2-like cells in the injured area, and the increase in the number of M2-like cells is not due to the natural course of SCI (Figures 4(c), 4(d)). We also used Western blotting to analyze the expression levels of these markers in the three groups quantitatively. The results verified the results of immunofluorescence (Figures 4(e)–4(g)). In short, limiting the number of Mets resulted in a significant increase in M2-like cells and a significant decrease in M1-like cells infiltrated in the injury.

In addition, we used RT-qPCR to detect the expression levels of marker proteins in M1/M2-like cells. In the SCI group, the mRNA expression levels of CD16, CD32, and iNOS were significantly increased. But they were decreased in the DNase l group (*P* < 0.05) (Figure 4(h)). For the mRNA expression levels of CD206, Arg-1, and YM1, the comparison results between the two groups were contrary to the above (Figure 4(i)). The quantitative analysis results of the M1 polarization product (IL-1*β*) and M2 polarization product (IL-10) reflected the same changes as above (Figure 4(j)).

To sum up, limiting the number of Mets increased the number of cells expressing the M2 phenotype and decreased the number of cells expressing the M1 phenotype. Meanwhile, it reduced the release of proinflammatory cytokines (IL-1*β*) and increased the release of anti-inflammatory cytokines (IL-10).

### 3.3. LL37 On Mets Can Interact with P2X7R to Activate the Downstream NF-*κ*B Proinflammatory Pathway

**Figure 5:**
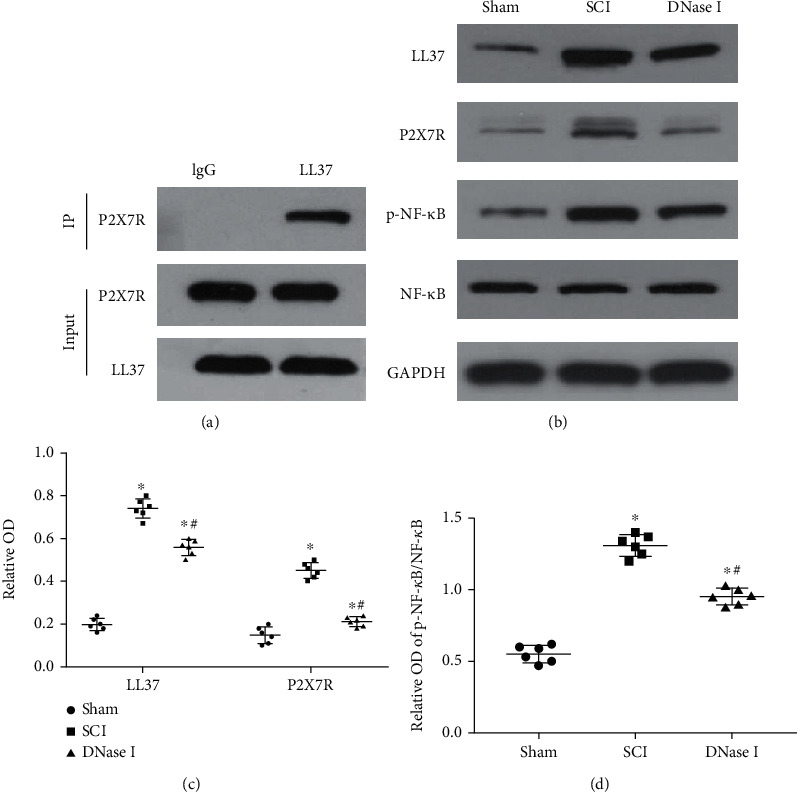
LL37 from Mets can activate the downstream NF-*κ*B pathway by interacting with P2X7R. (a) Representative images of Co-IP. Cell lysates of spinal cord injured tissues were immunoprecipitated with anti-LL37 and then immunoblotted with anti-P2X7R and anti-LL37. Rabbit anti-IgG was used as a negative control. Co-IP: Co-Immunoprecipitation. (b) Typical immunoblots of LL37, P2X7R, p-NF-*κ*B, and NF-*κ*B in the injured area of each group at 7 days after the operation. GAPDH was used as a loading control. (c, d) Scatterplot of the relative OD of LL37, P2X7R, and p-NF-*κ*B/NF-*κ*B in each group. Data are presented as Mean ± SD(n = 6).  ^∗^*P* < 0.05 vs. Sham group; #P < 0.05 vs. SCI group (one-way analysis of variance followed by the least significant difference test).

To further explore the possible mechanism of Mets in SCI, we used Co-IP to demonstrate that LL37, the specific structural component of Mets, could bind to P2X7R on the cell membrane of macrophage/microglia (Figure 5(a)). In addition, we also qualitatively and quantitatively analyzed the expression of LL37, P2X7R, NF-*κ*B, and p-NF-*κ*B in injured tissues of the three groups by Western blotting. The results showed that the expression levels of LL37, P2X7R, NF-*κ*B, and p-NF-*κ*B in the DNase I group were significantly lower than those in the SCI group, but higher than those in the Sham group (*P* < 0.05) (Figures 5(b)–5(d)).

### 3.4. The Concentration Levels of Cf-DNA, CD68, and CitH3 in Human Serum Can Help to Assess the Stage and Severity of SCI

**Figure 6:**
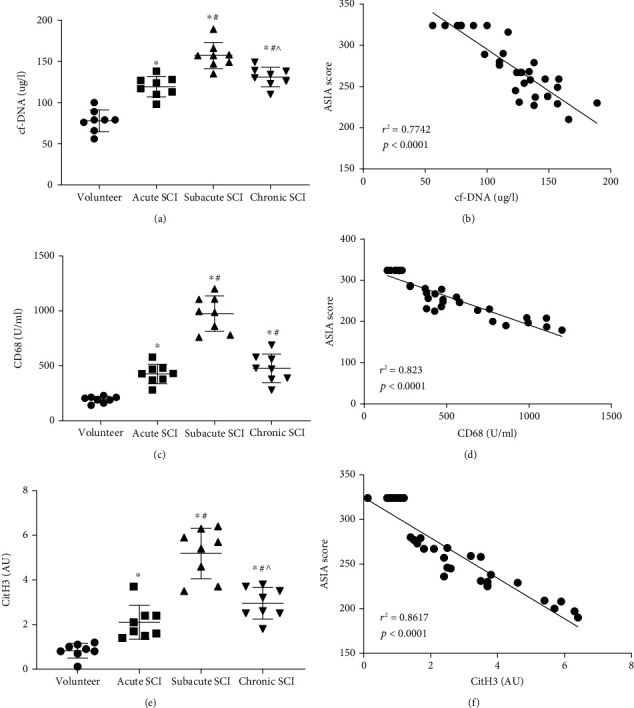
The concentrations of Mets-associated proteins in human serum can be used to evaluate SCI. Scatterplot of the concentrations of cf-DNA, CD68, and CitH3 in serum of healthy volunteers and patients at different SCI stages. Data are presented as Mean ± SD (*n* = 6).  ^∗^*P* < 0.05 vs. volunteer group; ^#^*P* < 0.05 vs. acute SCI group;  ^∧^*P* < 0.05 vs. subacute SCI group (one-way analysis of variance followed by the least significant difference test) (a, c, e). Linear regression analysis between ASIA score and the concentrations of cf-DNA, CD68, and CitH3 in serum of all subjects, respectively (b, d, f).*r*^2^ is the coefficient of determination. The value range of *r*^2^ is 0 to 1. *P* < 0.001 means a linear regression relationship between the *X* and *Y* variable.

In our study, we also detected the concentration levels of cf-DNA, CD68, and CitH3 in volunteers' and patients' serum. The difference between groups is consistent with the results that we previously detected in the rats' serum of the three groups (Figure S3–5). Furthermore, we subdivided the concentrations of these markers in the patient's serum according to the natural stage of the disease to find the relationship between them and the stage of SCI. Moreover, we briefly analyzed the relationship between them and ASIA scores, to find a simple and accurate method to evaluate the stage and severity of SCI. See Table S1 for more clinical experimental details. As shown in Figures 6(a), 6(c), and 6(e), the concentration of cf-DNA, CD68, and CitH3 in serum increased gradually from acute to the subacute stage after SCI, while the concentration of corresponding detectors decreased gradually from subacute to the chronic stage. The difference in each stage was statistically significant (*P* < 0.05) except for the comparison of CD68 levels between the subacute stage and chronic stage. Furthermore, we performed linear regression analysis between the concentration of cf-DNA, CD68, CitH3, and ASIA scores in the serum of healthy volunteers and patients. It can be seen that there is a negative correlation between the three detectors and ASIA scores, and the correlation is higher, the linear fitting degree is much better (Figures 6(b), 6(d), and 6(f)). In summary, we have reason to believe that there is a strong correlation between the concentration levels of cf-DNA, CD68, and CitH3 in human serum and ASIA scores, and the concentration of cf-DNA, CD68, and CitH3 in human serum can be used to accurately assess the stage and severity of SCI.

## 4. Discussion

Extracellular traps were first discovered in 2004. They were initially found to be released by neutrophils and associated with immune response [10]. Then, a lot of subsequent studies found that a variety of cells can also release similar structures to participate in local pathophysiological processes, such as eosinophils, basophils, mast cells, and monocytes/macrophages [25, 26]. Some studies have shown that the release of extracellular traps may be a new way of cell death [9]. This unique way of cell death exists in the pathophysiological process of many diseases, such as autoimmunity, and ischemia-reperfusion damage [27, 28]. Therefore, it has been widely studied. Nowadays, the research on Nets is extensive and in-depth, involving many fields such as infection, cardiovascular disease, autoimmune disease, and tumors [29]. However, there are few studies on Mets. Most of the related studies are limited to infection [15–17], autoimmune diseases [18, 19], and other fields. Our study focuses on the field of spinal cord injury (SCI). We explore the existence of Mets in the spinal cord injured area and its potential mechanism for the first time, which is a highlight of our study.

Previous studies have shown that there was a large number of neutrophils infiltration in the injured area in the early stage after SCI, and then the monocytes in the blood circulation migrated and infiltrated the injured area, differentiated into various types of macrophages, and acted as the main inflammatory cells in the injured area, leading local inflammation [30, 31]. In addition, some other studies also found that neutrophils infiltrated locally after SCI could release Nets [24], and these Nets could induce M1-like macrophages to release ETs-like structures [32]. Therefore, we boldly speculated that there may be Mets in the SCI area.

DNase I has been used as one of the effective means to degrade ETs for many years [11–13, 33]. It is a kind of deoxyribonuclease and can be used to directionally clear the DNA components in ETs' structure, thereby limiting the number of Mets [11]. Based on our hypothesis, we used DNase I as a grouping strategy for our research design. For this reason, we specially set up a group of rats to inject a certain amount of DNase I through the tail vein immediately after injury. At the beginning of the study, we first analyzed the relationship between Mets and the pathological environment of the injury, behavior, and electrophysiological changes by artificially reducing the number of Mets in the injured area. Ultimately, we demonstrated that the use of Mets degrading agent (DNase I) can reduce the apoptosis of nerve cells and shrink the nerve cavity in the injured area after SCI, to improve the local pathological environment and promote the recovery of nerve conduction and motor function. This also indirectly reflects the production of Mets after SCI and the positive contribution of Mets in aggravating the local inflammatory response of SCI.

Citrulline histone H3 (CitH3) is a posttranslational modified form of histone H3, which can be used to mark Mets [16]. CD68, a glycosylated type I membrane protein, is mainly expressed in the late endosomes and lysosomes of macrophages and can be used as a specific marker of macrophages [34]. The extracellular combination of the two markers can contributes to the specific recognition of extracellular trap structures derived from macrophages. In this study, we used immunofluorescence staining to recognize Mets and the cells that released them in the SCI area. Then, we further demonstrated the existence of macrophages that release extracellular traps by flow cytometry and observed the presence of Mets under the electron microscope. Based on the above results, we confirmed that Mets were produced locally after SCI, which verifies our hypothesis. In addition, these results can also reasonably explain our previous experimental results on the function and phenotype after SCI.

The microenvironment of SCI can induce macrophages and in-situ macrophages (microglia) to differentiate into M1- or M2-like cells [3, 31]. Among them, M2-like macrophages secrete protective factors and play an anti-inflammatory role, while M1-like macrophages secrete destructive factors and play a proinflammatory role [3, 35]. The two types of cells can be transformed into each other. Thus, it can be seen that inducing macrophages to M2 polarization is a therapeutic strategy to improve the persistent inflammation of SCI. In vitro stimulation test found that Mets could be produced by M1-like macrophages, but not by M2-like macrophages [32, 36], which means reducing the production of Mets may affect the expression of the M1 phenotype. In this study, we found that limiting the number of Mets could prevent local macrophage/microglia from expressing M1 phenotype and releasing proinflammatory factors such as IL-1*β*, and promote M2 phenotype expression and release anti-inflammatory factors such as IL-10, to improve the local inflammation in injured area. Thus, we thought that Mets may be an important reason for the continuous activation of M1-like macrophage/microglia under the pathological condition of SCI, and limiting the number of Mets may transform them into M2-like cells. Besides, some studies have shown that IL-10 could polarize macrophages to M2-like cells [37], which may form a virtuous circle of increasing the number of M2-like macrophages.

**Figure 7:**
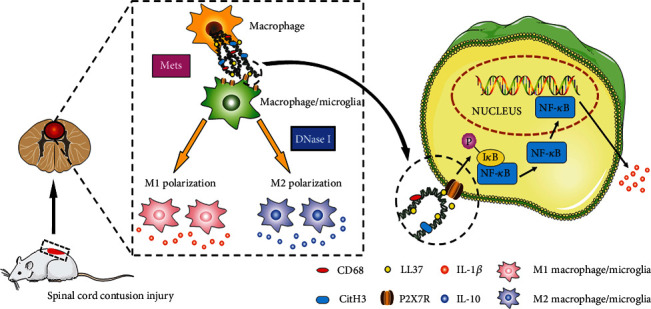
Interactions between macrophage infiltrated in the injured area and macrophage/microglia. 7 days after SCI, most of the locally infiltrated macrophages/microglia express the M1 phenotype and secrete a large number of proinflammatory factors such as IL-1*β*, which aggravate the local inflammation of the injury. In recent works, we found that macrophages infiltrated in the spinal cord injured area can release Met. It may be a key factor in the M1 polarization of macrophage/microglia. This happens through LL37 triggering downstream NF-*κ*B proinflammatory pathway via the activation of the P2X7R. However, limiting the number of Mets by using DNase I can promote the M2 polarization of macrophage/microglia and the release of anti-inflammatory factors such as IL-10. Therefore, Mets can be a new target for the treatment of SCI. SCI: spinal cord injury; IL: interleukin. Met: macrophage extracellular trap. P2X7R: P2X purinoreceptor 7.

Finally, we explored the possible mechanism of Mets in SCI. LL37 is an antimicrobial peptide and it can exist on extracellular traps [14]. Previous studies have found that LL37 can combine with P2X7R on the cell membrane surface to induce the proinflammatory differentiation of macrophages [14, 38]. P2X7R is a nonselective gated cation channel discovered in almost all immune cells. Previous studies have found that it could bind to corresponding ligands, activate downstream NF-*κ*B, and then induce M1 polarization, promote the release of inflammatory factors, and aggravate inflammatory response [39–41]. In our study, we demonstrated that LL37, the structural component of Met, could bind to P2X7R on the membrane of macrophages. Western blotting results showed that the expression of LL37, P2X7R, NF-*κ*B, and p-NF-*κ*B was upregulated in injured tissues. Based on the above results, we got a possible immune regulation mechanism. As shown in Figure 7, Mets may activate the downstream NF-*κ*B pathway through the interaction between its structural component LL37 and P2X7R on the cell membrane of macrophage/microglia, and then regulate the polarization of macrophage/microglia to M1 direction and release inflammatory factors such as IL-1*β*, leading to the continuous progression of inflammation. In addition, we thought that the presence of Mets in the local microenvironment may be one of the important factors leading to the continuous activation of M1-like cells and inflammatory cascade reaction.

ASIA scale is mainly used to evaluate the severity of SCI, and it has been the international gold standard for evaluating SCI [42]. It is worth noting that we also conducted a simple linear regression analysis of the relationship between Mets-related markers in human serum and ASIA scores in patients with SCI, and found a strong correlation between them. Besides, we also found a relationship between the concentration of cf-DNA, CD68, and CitH3 and the staging of SCI. In a word, these results illustrated that the concentration of cf-DNA, CD68, and CitH3 in serum can be used as a potential index to assess the severity of SCI, which may contribute to rapid diagnosis and accurate medical treatment in clinical practice.

However, there are also some shortcomings in this study. Firstly, as an in-vivo study, further in-vitro experiments are needed to verify this extracellular regulatory mechanism. Secondly, we only explored the mechanism of LL37/P2X7R/NF-*κ*B whether Mets can promote inflammation through other potential mechanisms remains to be studied. Finally, further in-vitro experiments need to be carried out to explore the difference in M1 polarization of macrophage and microglia induced by Mets, respectively.

In general, we demonstrated that infiltrating macrophages in the SCI area can release Mets, and then we explored the possible mechanism of Mets. Mets can activate the downstream NF-*κ*B pathway through the interaction between its structural component LL37 and P2X7R on the cell membrane of macrophage/microglia, and then promote the continuous activation of M1-like cells and the release of proinflammatory factors, thus resulting in the progression of persistent inflammation. However, limiting the number of Mets may reverse this change, promote the polarization of macrophages/microglia to the M2 direction and the release of anti-inflammatory factors, thus reducing the pathological manifestations such as tissue injury, cell death, and cavity formation caused by inflammation and promoting the recovery of nerve conduction and motor function of the spinal cord. In addition, we also found a serological index that can be used to assess the degree and stage of SCI, thus providing a new idea for the reasonable evaluation of the severity, stage, and motor rehabilitation of spinal cord injury.

## 5. Conclusion

In this study, we demonstrated that infiltrating macrophages in the SCI area can release Mets, and then we explored the possible mechanism of Mets. Mets can activate the downstream NF-*κ*B pathway through the interaction between its structural component LL37 and P2X7R on the cell membrane of macrophage/microglia, and then promote the continuous activation of M1-like cells and the release of proinflammatory factors, thus resulting in the progression of persistent inflammation. Besides, we also found a serological index that can be used to assess the degree and stage of SCI. In short, our study may provide a new target for the treatment of local inflammation of secondary SCI and a new index for clinical evaluation of the severity of SCI.

## Figures and Tables

**Table 1:** Sequences of primers for RT-qPCR.

Gene	Forward primer sequence (5′-3′)	Reverse primer sequence (5′-3′)
CD16	TTTGGACACCCAGATGTTTCAG	GTCTTCCTTGAGCACCTGGATC
CD32	AATCCTGCCGTTCCTACTGATC	GTGTCACCGTGTCTTCCTTGAG
iNOS	GGTGAAGGGACTGAGCTGTT	ACGTTCGTTCTCTTGCA
Arg-1	CACCTGAGCTTTGATGTCG	TGAAAGGAGCCCTGTCTTG
YM1	GAGGTAATGAGTGGGTTGG	ACGGCACCTCCTAAATTGT
CD206	AAGGAAGGTTGGCATTTGT	CTTTCAGTCCTTTGCAAGC
GAPDH	GCCAAGGCTGTGGGCAAGGT	TCTCCAGGCGGCACGCAGA

## Data Availability

The data used to support the findings of this study are available from the corresponding author upon request.

## Abbreviations

SCI: Spinal cord injury
ET: Extracellular trap
BMDM: Bone marrow-derived macrophage
Net: Neutrophil extracellular trap
Met: Macrophage extracellular trap
CitH3: Citrullinated histone H3
P2X7R: P2X purinoreceptor 7
IL-1*β*: Interleukin-1*β*
IL-10: Interleukin-10.

## Supplementary Materials

Supplementary 1 Table S1: baseline characteristics of the participants.

Supplementary 2 Table S2: inclusion and exclusion criteria of subjects.

Supplementary 3 Figure S1: macrophages and Mets under TEM. In this picture, the extracellular DNA structure was released from the macrophage and extended outside the cell membrane. ∗: macrophage; #: Met. *N* = 3*n*; *n* = 2 (2 rats in each group). Scale bar: 5.0 *μ*m. Mets: macrophage extracellular traps; TEM: transmission electron microscope.

Supplementary 4 Figure S2: experimental design and grouping. (a) BBB scale and inclined plate test were performed at 0, 1, 7, 14, 21, and 28 days after SCI. Footprint analysis was performed on day 7 after SCI. Besides, an EMG monitor was performed to observe the nerve conduction on day 7 after SCI. BBB: Basso-Beattie-Bresnahan; EMG: electromyographic. (b) To explore the effect of Mets on the local pathological environment, H&E, Nissl, and TUNEL staining were performed on day 7 after SCI. SCI: spinal cord injury; Met: macrophage extracellular trap. (c) Immunofluorescence staining, Flow cytometry, and Western blotting were performed on day 7 after SCI to detect Mets in the injured area. (d) Immunofluorescence staining, Western blotting, and RT-qPCR were performed on day 7 after SCI to explore the effect of limiting the number of Mets on M1/M2 polarization of macrophage/microglia. (e) Co-IP was performed on day 7 after SCI to explore the interaction between LL37 and P2X7R. Additionally, Western blotting was performed on day 7 after SCI to evaluate the combination of LL37 and P2X7R in regulating the NF-*κ*B pathway. P2X7R: P2X purinoreceptor 7. (f) To explore the relationship between the concentration of Mets structural proteins in human serum and the stage and ASIA score of SCI, ELISA, and ASIA scales were performed at acute, subacute, and chronic stages of SCI, respectively. ELISA: enzyme-linked immunosorbent assay. Notes: The time of sampling and the number of rats in each group are indicated in brackets behind. The same color represents the same rats used in these methods. In addition, all the rats were randomly divided into three groups: Sham group (*n* = 32), SCI group (*n* = 32), and DNase I group (*n* = 32). Rats in DNase I group were injected with DNase I via tail vein immediately after SCI. Healthy volunteers and SCI patients were divided into two groups: volunteer group (*n* = 8), SCI group (*n* = 24) (including acute stage (*n* = 8), subacute stage (*n* = 8), and chronic stage (*n* = 8)).

Supplementary 5 Figure S3–5: comparison of CD68, cf-DNA, and CitH3 in rats' serum of the three groups. Typical bar graphs of the concentrations of CD68, cf-DNA, and CitH3 in rats' serum of the three groups, respectively. The concentrations of the three markers in the DNase I group is lower than those in the SCI group but higher than those in the Sham group. Data are presented as Mean ± SD (*n* = 6).  ^∗^*P* < 0.05 vs. Sham group; #*P* < 0.05 vs. SCI group.
