# Supplementary material for: Macrophage Extracellular Traps Exacerbate Secondary Spinal Cord Injury by Modulating Macrophage/Microglia Polarization via LL37/P2X7R/NF-κB Signaling Pathway
Source: Oxid Med Cell Longev. 2022 Nov 23;2022:9197940. doi: 10.1155/2022/9197940 (PMC9713475; doi:10.1155/2022/9197940)
Supplement: Supplementary 1 — Table S1: baseline characteristics of the participants. [file 9197940.f1.docx]

**Table S1. Baseline characteristics of the participants**

| Characteristic | Healthy Controls (n=8) | SCI Patients (n=24) | *p* Value |
| --- | --- | --- | --- |
| Age, year, mean ± SD | 39.83 ± 13.13 | 41.96 ± 12.76 | 0.6875^a^ |
| Gender, male/female, n | 4/4 | 13/11 | >0.9999^b^ |
| Cause of injury, n (%) |  |  |  |
| Motor vehicle accident | NA | 12 (50.00%) | - |
| Falls | NA | 8 (33.33%) | - |
| Other | NA | 4 (16.67%) | - |
| ASIA score, mean ± SD | 324 ± 0 | 256.86 ±54.32 | <0.0001^a^ |
| Severity of initial neurological deficit |  |  |  |
| ASIA Impairment Scale A | NA | 3 (12.50%) | - |
| ASIA Impairment Scale B | NA | 8 (33.33%) | - |
| ASIA Impairment Scale C | NA | 10 (41.67%) | - |
| ASIA Impairment Scale D | NA | 3 (12.50%) | - |
| Tetraplegia | NA | 5 (20.83%) | - |
| Length of stay in ICU, days |  |  |  |
| 0–2 | NA | 10 (41.67%) | - |
| 3–14 | NA | 8 (33.33%) | - |
| ≥15 | NA | 6 (25.00%) | - |
| Length of stay in acute care, mean ± SD | NA | 24 ± 23 | - |
| SCIM upon admission to rehabilitation, mean ± SD | NA | 24 ± 21 | - |
| SCIM upon discharge from rehabilitation, mean ± SD | NA | 57 ± 25 | - |

Note: ^a^Analysed by Independent sample t-test; ^b^Analysed by Chi-square test.

Abbreviations: SCI, spinal cord injury; SD, standard deviation; ASIA, American Spinal Cord Injury Association; ICU, intensive care unit; SCIM, Spinal Cord Independence Measure.
