# Supplementary material for: Macrophage Extracellular Traps Exacerbate Secondary Spinal Cord Injury by Modulating Macrophage/Microglia Polarization via LL37/P2X7R/NF-κB Signaling Pathway
Source: Oxid Med Cell Longev. 2022 Nov 23;2022:9197940. doi: 10.1155/2022/9197940 (PMC9713475; doi:10.1155/2022/9197940)
Supplement: Supplementary 2 — Table S2: inclusion and exclusion criteria of subjects. [file 9197940.f2.docx]

**Table S2:** **Inclusion and exclusion criteria of subjects**

| **1. Patients with spinal cord injury:** |
| --- |
| **Inclusion criteria:** |
| （1）Age ≥ 18 years old; |
| （2）The patients were identified as having simple spinal cord injury; |
| （3）The injured segments include the cervical, thoracic, and lumbar spinal cord; |
| **Exclusion criteria:** |
| （1）Age <18 years; |
| （2）Acute exacerbation of subacute or chronic spinal cord injury, such as acute spinal cord injury caused by external force in patients with spinal cord disease; |
| （3）Cases of cauda equina nerve or nerve root injury; |
| （4）Cases complicated with spinal cord injury or infection at other sites; |
| （5）Cases of partial or complete disconnection of the spinal cord, such as firearm injury and knife stabbing injury; |
| （6）Patients with traumatic brain injury, severe chest injury, abdominal organ injury, and severe limb and pelvic fractures; |
| （7）Functional injures such as spinal cord shock and spinal cord concussion; |
| （8）Combined with other diseases that can lead to the increase of cf-DNA, CD68, and CitH3 protein, such as brain infectious diseases, brain or spinal cord tumors, small cell lung cancer, Alzheimer's disease, mental system diseases, and other cases; |
| （9）Unable to conduct complete follow-up; |
| **2. Healthy volunteers:** |
| **Inclusion criteria:** |
| （1）Age ≥ 18 years old; |
| （2）Healthy without basic diseases or medical histories such as hypertension and diabetes; |
| （3）Informed consent to the study and voluntary participation; |
| **Exclusion criteria:** |
| （1）Previous history of the spinal cord or brain injury and surgical treatment; |
| （2）Previous history of the nervous system, such as cerebral hemorrhage, cerebral infarction, epilepsy, Parkinson's disease, Alzheimer's disease, Guillain Barre syndrome, etc; |
| （3）Previous psychiatric diseases, such as schizophrenia, depression, anxiety, etc; |
| （4）Previous history of alcohol, drug or drug abuse; |
| （5）Poor cooperation; |
