# Supplementary figures and images for: Macrophage Extracellular Traps Exacerbate Secondary Spinal Cord Injury by Modulating Macrophage/Microglia Polarization via LL37/P2X7R/NF-κB Signaling Pathway
Source: Oxid Med Cell Longev. 2022 Nov 23;2022:9197940. doi: 10.1155/2022/9197940 (PMC9713475; doi:10.1155/2022/9197940)

## Slide 1
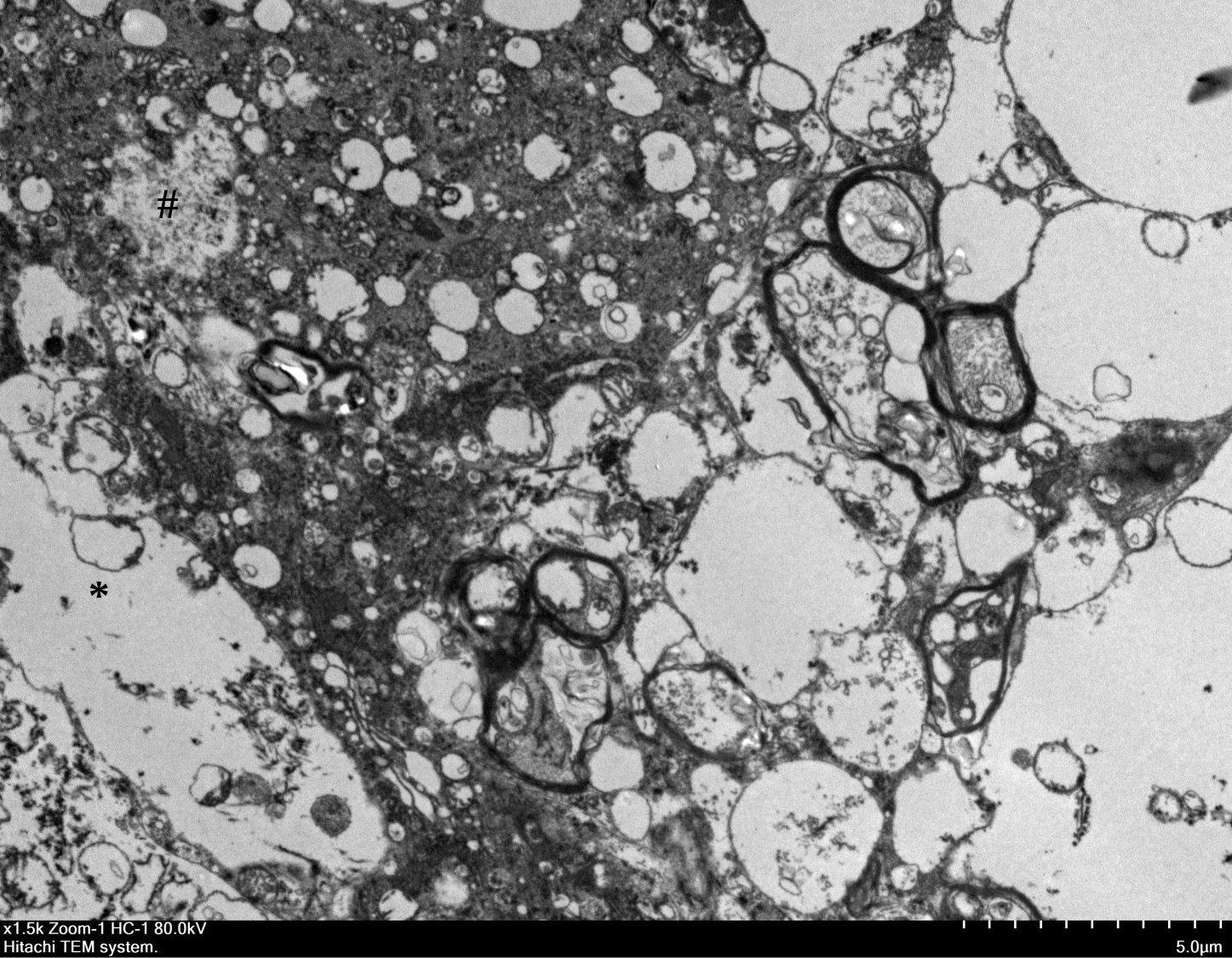

#
*

Supplement: Supplementary 3 — Figure S1: macrophages and Mets under TEM. In this picture, the extracellular DNA structure was released from the macrophage and extended outside the cell membrane. ∗: macrophage; #: Met. N = 3n; n = 2 (2 rats in each group). Scale bar: 5.0 μm. Mets: macrophage extracellular traps; TEM: transmission electron microscope. [file 9197940.f3.pptx]

## Slide 1
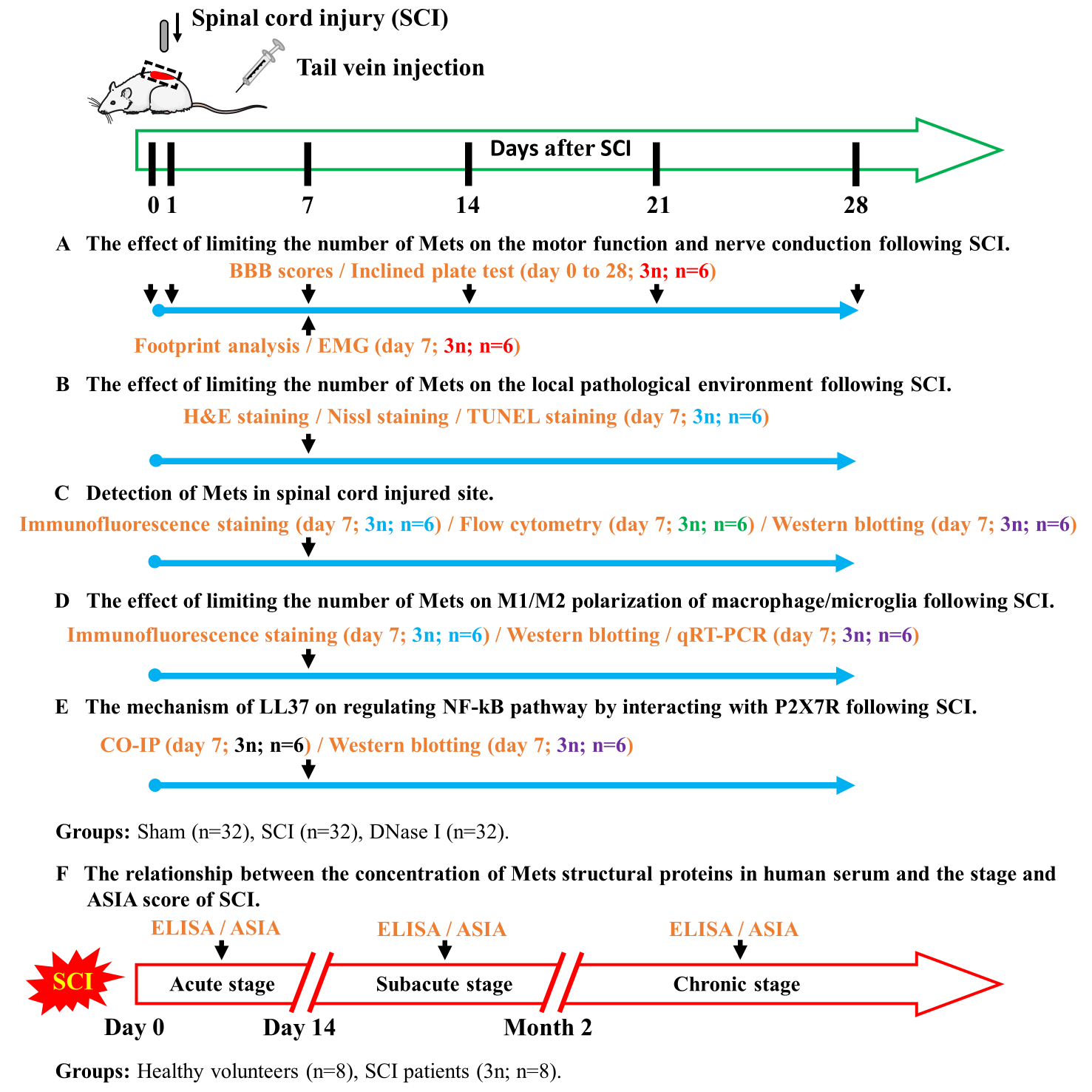

Supplement: Supplementary 4 — Figure S2: experimental design and grouping. (a) BBB scale and inclined plate test were performed at 0, 1, 7, 14, 21, and 28 days after SCI. Footprint analysis was performed on day 7 after SCI. Besides, an EMG monitor was performed to observe the nerve conduction on day 7 after SCI. BBB: Basso-Beattie-Bresnahan; EMG: electromyographic. (b) To explore the effect of Mets on the local pathological environment, H&E, Nissl, and TUNEL staining were performed on day 7 after SCI. SCI: spinal cord injury; Met: macrophage extracellular trap. (c) Immunofluorescence staining, Flow cytometry, and Western blotting were performed on day 7 after SCI to detect Mets in the injured area. (d) Immunofluorescence staining, Western blotting, and RT-qPCR were performed on day 7 after SCI to explore the effect of limiting the number of Mets on M1/M2 polarization of macrophage/microglia. (e) Co-IP was performed on day 7 after SCI to explore the interaction between LL37 and P2X7R. Additionally, Western blotting was performed on day 7 after SCI to evaluate the combination of LL37 and P2X7R in regulating the NF-κB pathway. P2X7R: P2X purinoreceptor 7. (f) To explore the relationship between the concentration of Mets structural proteins in human serum and the stage and ASIA score of SCI, ELISA, and ASIA scales were performed at acute, subacute, and chronic stages of SCI, respectively. ELISA: enzyme-linked immunosorbent assay. Notes: The time of sampling and the number of rats in each group are indicated in brackets behind. The same color represents the same rats used in these methods. In addition, all the rats were randomly divided into three groups: Sham group (n = 32), SCI group (n = 32), and DNase I group (n = 32). Rats in DNase I group were injected with DNase I via tail vein immediately after SCI. Healthy volunteers and SCI patients were divided into two groups: volunteer group (n = 8), SCI group (n = 24) (including acute stage (n = 8), subacute stage (n = 8), and chronic stage (n = 8)). [file 9197940.f4.pptx]

## Slide 1
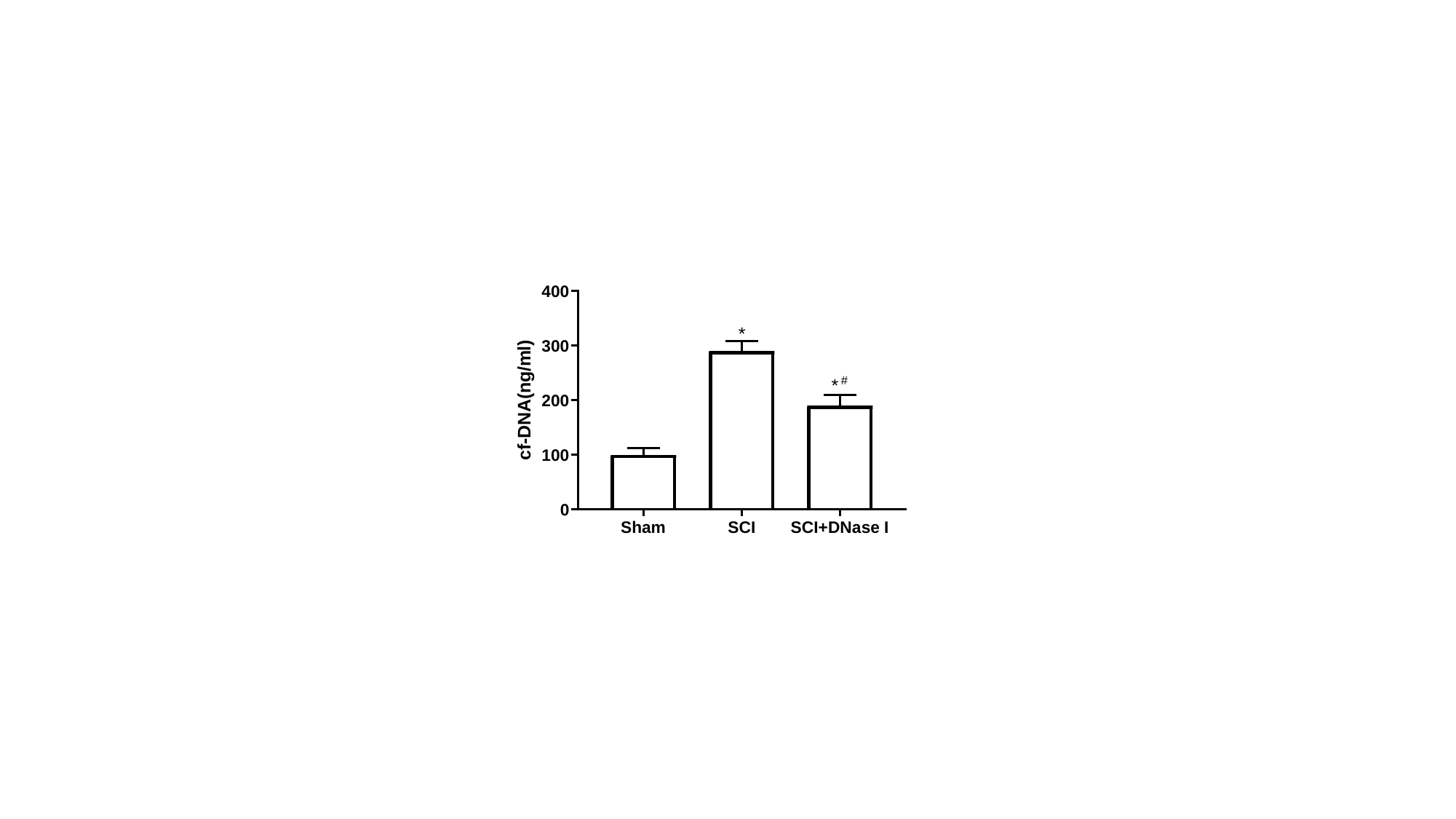

## Slide 2
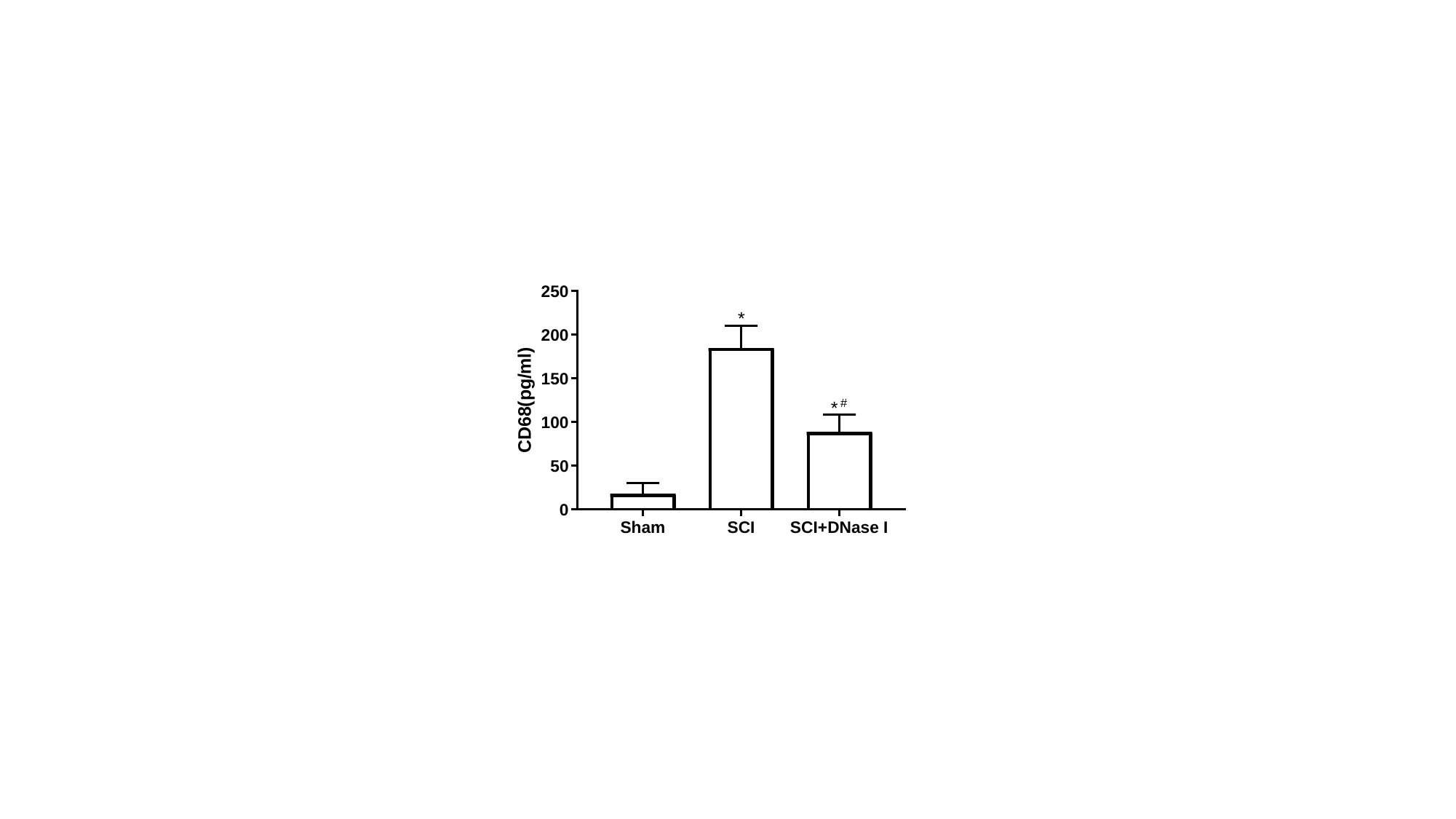

## Slide 3
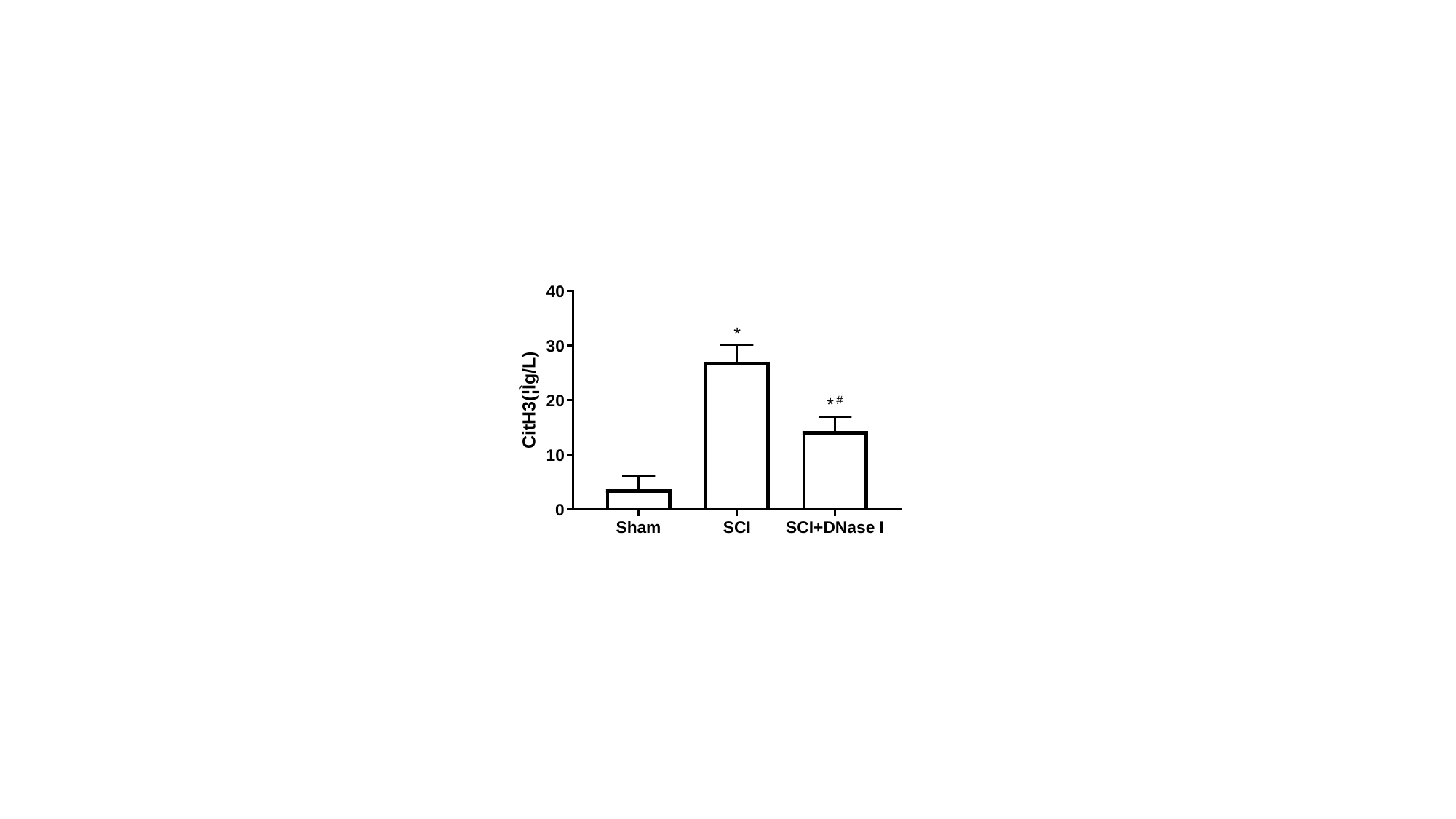

Supplement: Supplementary 5 — Figure S3–5: comparison of CD68, cf-DNA, and CitH3 in rats' serum of the three groups. Typical bar graphs of the concentrations of CD68, cf-DNA, and CitH3 in rats' serum of the three groups, respectively. The concentrations of the three markers in the DNase I group is lower than those in the SCI group but higher than those in the Sham group. Data are presented as Mean ± SD (n = 6). ∗P < 0.05 vs. Sham group; #P < 0.05 vs. SCI group. [file 9197940.f5.pptx]
